# A TRIzol-based method for high recovery of plasma sncRNAs approximately 30 to 60 nucleotides

**DOI:** 10.1038/s41598-022-10800-0

**Published:** 2022-04-26

**Authors:** Kristen P. Rodgers, Alicia Hulbert, Hamza Khan, Maria Shishikura, Shun Ishiyama, Malcolm V. Brock, Yuping Mei

**Affiliations:** 1 The Sidney Kimmel Comprehensive Cancer Center, Johns Hopkins School of Medicine, 1650 Orleans Street, Baltimore, MD 21287 USA; 2 Department of Surgery, University of Illinois at Chicago College of Medicine, Chicago, IL USA

**Keywords:** Biological techniques, Biotechnology, Molecular biology

## Abstract

Protein functional effector sncRNAs (pfeRNAs) are approximately 30–60 nucleotides (nt), of which the extraction method from plasma has not yet been reported. Silver staining in a high-resolution polyacrylamide gel suggested that the majority of plasma sncRNAs extracted by some broadly used commercial kits were sncRNAs from 100 nt upwards. Additionally, TRIzol’s protocol is for long RNA but not sncRNA recovery. Here, we report a TRIzol-based frozen precipitation method (TFP method), which shows rigor and reproducibility in high yield and quality for plasma sncRNAs approximately 30–60 nt. In contrast to the yields by the commercial kit, plasma sncRNAs extracted by the TFP method enriched more sncRNAs. We used four different pfeRNAs of 34 nt, 45 nt, 53 nt, and 58 nt to represent typical sizes of sncRNAs from 30 to 60 nt and compared their levels in the recovered sncRNAs by the TFP method and by the commercial kit. The TFP method showed lower cycle threshold (CT) values by 2.01–9.17 cycles in 38 plasma samples from 38 patients, including Caucasian, Asian, African American, Latin, Mexican, and those who were a mix of more than one race. In addition, pfeRNAs extracted by two organic-based extraction methods and four commercial kits were undetermined in 22 of 38 samples. Thus, the quick and unbiased TFP method enriches plasma sncRNA ranging from 30 to 60 nt.

## Introduction

Circulating cell-free RNAs from plasma or serum mainly consist of small non-coding RNAs (sncRNAs). Effectively extracting these sncRNAs is important for downstream applications. However, different types of sncRNAs have different features, such as length, modification, tertiary structure, etc. Thus, a specific type of sncRNA may need a unique and effective extraction method based on its features.

Of the circulating cell-free sncRNAs in plasma or serum^1,2^, miRNA and its precursor (pre-miRNA), piwi-interacting RNA (piRNA), and protein functional effector sncRNA (pfeRNA) are major subpopulations associated with tumorigenesis and cancer development. These sncRNAs could serve as non-invasive biomarkers for diagnosing, detecting, and monitoring malignant disease and the therapeutic response of patients^3–7^.

The mature miRNA is ~ 22 nt, its pre-miRNA is above 60 nt^8–12^, piRNA is around 21–34 nt^5,13–16^, and pfeRNA is approximately 30–60 nt^4,7,16^. To date, plasma extraction methods for miRNAs and their precursors as well as piRNAs have been well documented. These extraction methods may broadly be grouped into organic extraction, filtered-based methods, and magnetic particle-based methods^18^. A popular choice is a filter-based method, such as the miRNeasy Serum/Plasma Kit (Qiagen)^18^, a commercial kit for the extraction of plasma sncRNA.

The broadly used miRNeasy Serum/Plasma Kit is designed to purify total cell-free RNA, including primary miRNA and other small RNA from serum or plasma. However, we tried two methods based on organic extraction and four commercial kits, and found it hard to measure plasma pfeRNA, which is approximately 30–60 nt, in the yields by the commercial kits. Additionally, while TRIzol is a common reagent for RNA extraction, its instructions are not for sncRNA recovery. We thus optimized a method that is highly effective and productive in extracting plasma sncRNA ranging from 30 to 60 nt.

## Results

### Principle and workflow of the optimized TFP method for plasma pfeRNA ranging from 30–60 nt

**Figure 1:**
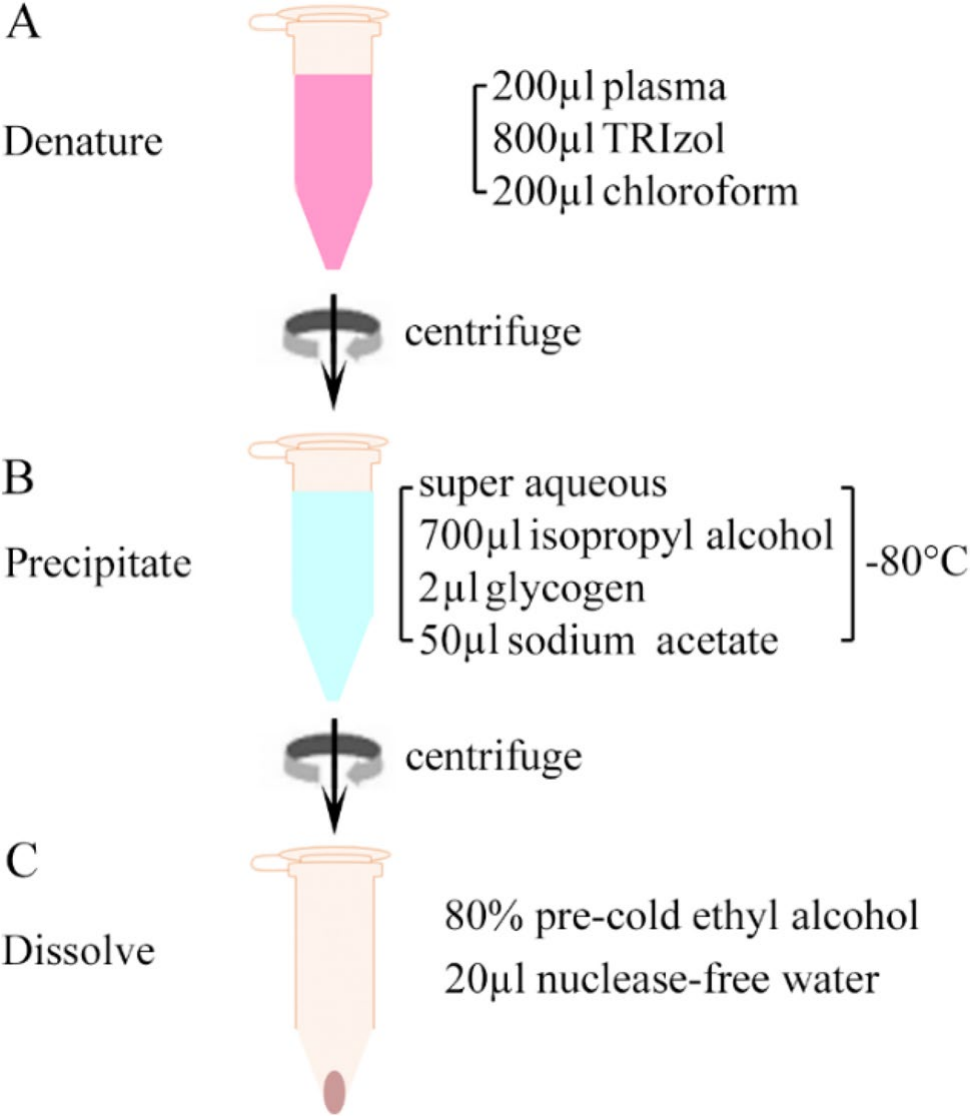
The principle and workflow of the optimized TFP method with specific reagents in the denaturing (**A**), precipitation (**B**), and washing/dissolving (**C**) steps.

Our optimized TFP method included denaturation, precipitation, and dissolving steps (Fig. 1). The TFP method combined phenol-based lysis at room temperature and glycogen-based precipitation at 4 °C. Specifically, 800 µl TRIzol Reagent was added to 200 µl of plasma, vortexed for 15 s at high speed, and kept at room temperature for 10 min. Then 200 µl chloroform was added to the mixture, vortexed for 15 s, and kept at room temperature for another 10 min (Fig. 1A). The sample was centrifuged at 12,000*g* for 10 min at 4 °C, and the super aqueous was transferred to a new tube. Then, 700 µl isopropyl alcohol, 2 µl glycogen, and 50 µl 3 M sodium acetate were added to the new tube and was mixed well. The tube was kept at − 80 °C until it was frozen (~ 25 min) (Fig. 1B). Next, the tube was centrifuged at 20,000*g* for 20 min at 4 °C. Finally, the white pellet was washed with 1000 µl 80% pre-cold ethyl alcohol and dissolved in 20 µl nuclease-free water (Fig. 1C). We found that 10 min was enough in the denaturing step, and prolonged incubation was unnecessary. In the precipitation step, we used both glycogen and sodium acetate to improve sncRNA recovery (Supplementary Fig. S1), and the frozen step enriched more plasma sncRNA. We used 80% ethyl alcohol to wash away the salts or any organic residues in the washing step while keeping sncRNAs.

### Distribution of sncRNAs extracted by the TFP method and by the commercial kit

**Figure 2:**
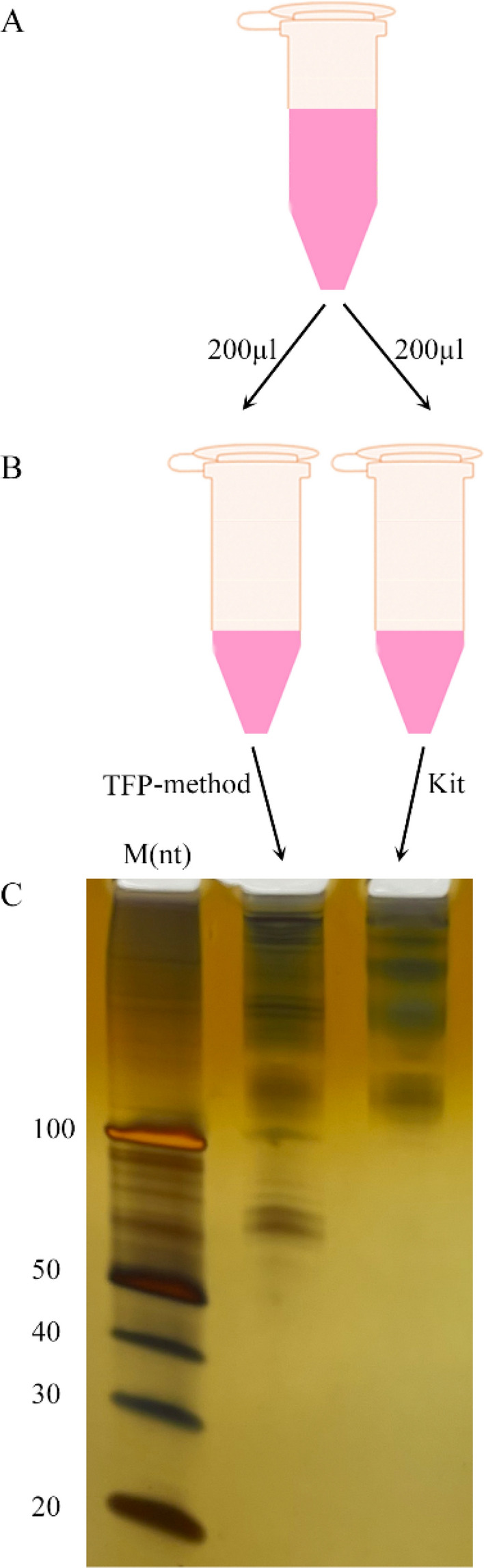
The extracted sncRNA distribution in the high-resolution gel was determined by silver staining. (**A**) The plasma from the same patient was mixed thoroughly before separation. (**B**) sncRNAs from 200 µl plasma were extracted by the TFP method and the kit separately. (**C**) The distribution of the yields extracted by the two methods. The full-length gel in (**C**) is presented in Supplementary Fig. S6, and the (**C**) was cropped from Lanes 1, 2, and 3 from the same gel.

Given that the miRNeasy Serum/Plasma Kit is a popular choice^18^ and that the advanced kit shows the best results (on Qiagen’s official website), we used the advanced kit for comparison. To compare objectively, we used the plasma of the same patient and mixed it thoroughly before separation (Fig. 2A). Strictly following the kit’s instructions, we used 200 µl plasma for extraction and 20 µl nuclease-free water for elution, and used the same corresponding volume for the TFP method (Fig. 1, Fig. 2A, B). We loaded 10 µl yields in a 15% denaturing polyacrylamide gel and performed silver staining. As shown in Fig. 2C, lane 2, plasma sncRNAs extracted by the TFP method included sncRNAs less than 100 nt. In contrast, plasma sncRNAs extracted by the commercial kit were sncRNAs from 100 nt upwards (Fig. 2C, lane 3). Also, we compared RNA yields by the TFP method with two methods based on organic extraction and another three commercial kits. Results showed that the TFP method recovered more sncRNAs compared to yields by every one of these five methods and kits (Supplementary Fig. S2).

### qPCR features of pfeRNA targets extracted by the TFP method and by two methods based on organic extraction and four commercial kits

To compare the quantity and quality of yields generated by these two methods, we first used 32 plasma specimens from 32 patients, including five patients with benign lung nodules and 27 patients with stage I/II non-small cell lung cancer (NSCLC) (Supplementary Table S1). All peripheral blood was collected before the patients underwent surgical resection or treatment. These patients included Caucasian, Asian, African American, Latin, Mexican, and those who were a mix of more than one race (Supplementary Table S1). Both genders, smokers, and non-smokers were included among the patients (Supplementary Table S1). Patients with benign lung nodules presented to their physicians with a CT or LDCT scanned image of a lung nodule that was later biopsy-proven to be benign, and patients with early-stage NSCLC were found free of any other cancer.

**Figure 3:**
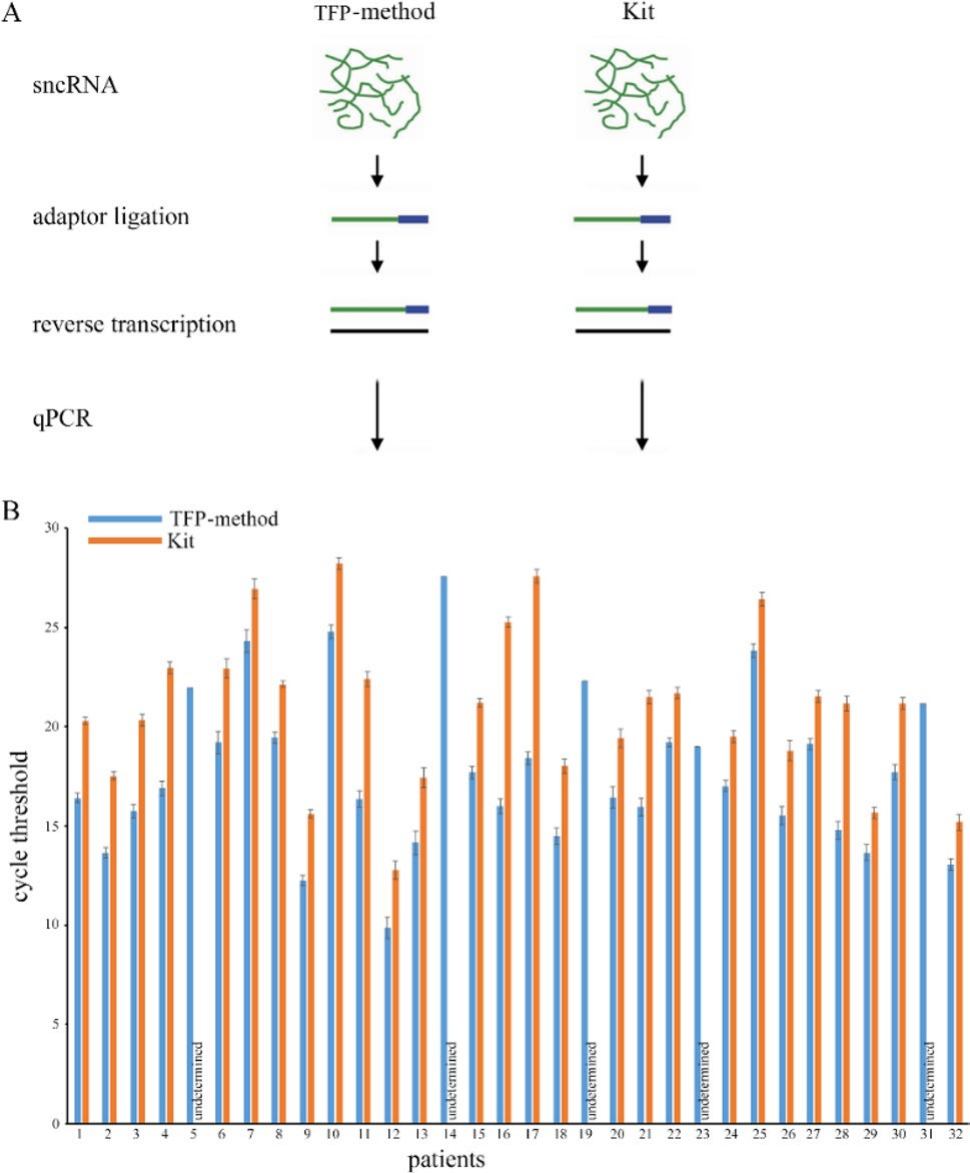
The steps for ligation and RT, and CT values of pfeRNA of 45 nt. (**A**) Five microliters of the extracted sncRNAs were ligated with adaptors in the 3′ end, and cDNA was synthesized by reverse transcription. (**B**) The specific CT values of pfeRNA of 45 nt from 32 clinical specimens. Three microliters of the synthesized cDNA, in a total volume of 20 µl, was used for qPCR detection. Undetermined means the levels of the target could not be detectable. All values are averages of six CT values from three independent repeats, and the error bars reflect the mean s.d.

**Figure 4:**
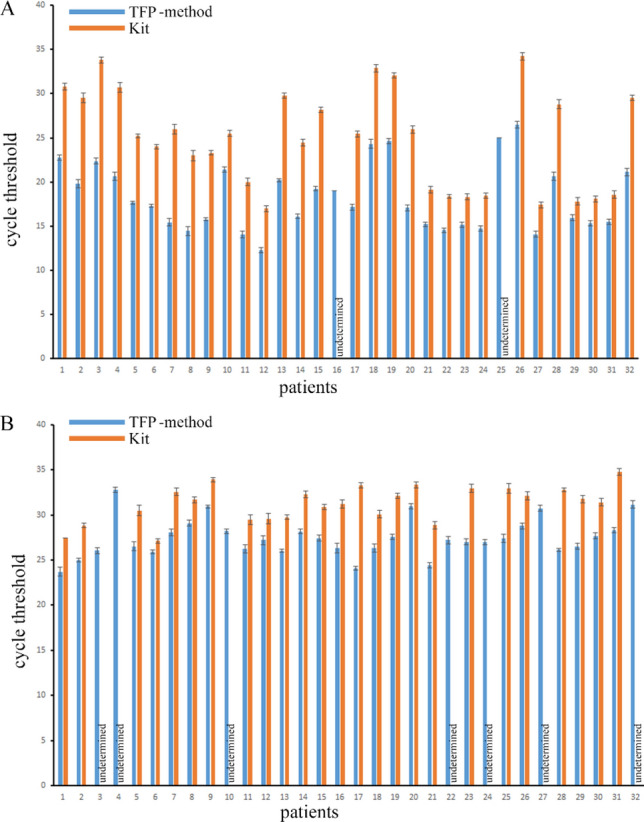
The specific CT values of pfeRNA of 34 nt (**A**) and 53 nt (**B**) from 32 clinical specimens. Three microliters of the synthesized cDNA, in a total volume of 20 µl, was used for qPCR detection. Undetermined means the levels of the target could not be detectable. All values are averages of six CT values from three independent repeats, and the error bars reflect the mean s.d.

Because pfeRNAs are too short to be amplified directly, we used an adaptor ligated to the 3’ end and performed reverse transcription (RT) (Fig. 3A; Supplementary Table S2). We tested each target in duplicate by qPCR and repeated each experiment three independent times in 32 clinical samples, and we thus obtained six CT values for each target. As shown in Fig. 3B, the CT values of pfeRNA with 45 nt by the TFP method were significantly lower than those by the commercial kit in all 32 clinical samples. Also, the target by the TFP method was detectable in each sample, and in contrast, the target by the commercial kit was undetectable in 5 of 32 samples (Fig. 3B). Two others of different pfeRNAs also demonstrated similar results in the yields by the commercial kit. Two samples exhibited undetermined pfeRNA of 34 nt (Fig. 4A), and seven samples exhibited undetermined pfeRNA of 53 nt (Fig. 4B). However, both targets were detectable in all 32 yields from 32 plasma specimens by the TFP method (Fig. 4). Also, the specific CT values of pfeRNA of 34 nt (Supplementary Fig. S3A) and 58 nt (Supplementary Fig. S3B) from six clinical specimens (Supplementary Table S1) demonstrated that the TFP method recovered more sncRNAs than these two organic-extraction methods and another three commercial kits. Consistent with reports on variable yields depending on the commercial kits^19^, our results showed that commercial kits recovered variable yields (Supplementary Figs. S2 and S3).

The amplification curves and melting curves were similar between the TFP method (Supplementary Figs. S4A and S5A) and the commercial kit (Supplementary Figs. S4B and S5B). However, some curves were not perfect in the yields by the commercial kit (Supplementary Figs. S4B and S5B), which could be caused by low quantity.

### Quantitative control of the comparison between these methods and kits

We strictly controlled the quantity of total sncRNAs for RT and the template volumes for QuantStudio PCR. We used 20 µl nuclease-free water to dissolve sncRNAs extracted from 200 µl plasma, 5 µl yields of 20 µl sncRNAs for RT, and cDNA in 20 µl solution after RT. We then used a 3 µl-cDNA template for each reaction. We implemented this quantity-control approach for two reasons. First, it generates similar CT values of the three independent repeats, and the variation was below 0.5 between the six CT values of each target (Figs. 3, 4). Second, it provides a rationale and objective comparative analysis.

## Discussion

### TFP method enriches sncRNAs ranging from 30 to 60 nt

We developed the TFP protocol based on the TRIzol-denatured method, and our results suggested that the TFP method recovers sncRNAs ranging from 30 to 60 nt. In contrast to TRIzol’s protocol for long RNA extraction, the advantage of TFP is that TFP enriches sncRNAs. Compared to the column-binding methods, the sncRNA enrichment by TFP could be caused by: First, the binding chance is higher for longer sncRNAs than shorter ones, and there is a possibility that not all shorter sncRNAs bind to the column compared to the longer ones, as the solution pass through the column in a high speed by centrifuge; Second, the washing by centrifuge could bring away some of the shorter sncRNAs that could not bind tightly; and the Third, not all bound shorter sncRNAs could be eluted. Additionally, we optimized reagent combination for sncRNA enrichment, for example, glycogen and low temperature for sncRNA precipitation and 80% ethyl alcohol for washing away the salts or any organic materials while keeping sncRNAs.

### A particular type of sncRNA may need a unique extraction method based on its specific features

Except for a shared feature of no protein production, different types of sncRNAs have different features, such as length and modification, with or without hairpins, etc*.*^4,5,20–24^*.* First, primary sequences and possible intramolecular base pairing form different secondary structures. Second, tertiary structural and corelative effects incorporate high dimensional features. And third, modifications can occur on the base, ribose, or both. These features could make different types of sncRNAs need different recovery methods. In this manuscript, the silvering staining, CT values, melting curves, and amplification curves of yields by the TFP method exhibited better quantity and quality in sncRNAs ranging from 30 to 60 nt compared to that extracted by the commercial kit. We propose a novel method for recovering pfeRNA, a specific type of sncRNA, from plasma for the first time.

## Materials and methods

### Patients and ethics declarations

We used 38 plasma samples from 38 patients, including patients with benign pulmonary nodules and patients with stage I/II NSCLC. The clinical biospecimens were from the Johns Hopkins Lung Cancer Specialized Program of Research Excellence (SPORE) Baltimore, MD, and the University of Illinois at Chicago Hospital Health Science System (UIHHSS, Chicago, IL, USA).

The study was approved by the Institutional Review Board (IRB) of Johns Hopkins University (JHU) and IRB of the University of Illinois at Chicago (UIC). The approval documents were NA_00005998 for JHU and IRB #2017-1286 and #2018-0755 for UIC. All procedures performed in our study involving human participants were in accordance with the ethical standards of the national research committee and with the 1964 Helsinki Declaration and its later amendments. All experimental protocols were approved prior to study initiation. Informed consent was obtained from each patient, and peripheral blood was collected after informed consent was obtained and prior to the patients undergoing surgical resection or with any treatment.

### Plasma preparation

Whole peripheral blood (7.5 ml) was collected in an anticoagulant tube (K_2_EDTA) and poured very slowly into a 15 ml conical tube with 5 ml Ficoll-Paque PLUS buffer (Millipore-Sigma, Cat #GE17-1440-02). The layered mixture was centrifuged for 10 min at 3000 rpm at 4 °C, and then the top plasma layer was transferred to 1.5 ml tubes. The samples should not be processed if the red cells had been lysed.

### Reagents and machines for RNAs extracted from plasma

TRIzol reagent (Thermo Fisher Scientific, cat# 15596018), TRIzol LS Reagent (Ambion, Cat# 10296010), chloroform (Sigma-Aldrich, Cat No. C2432), glycogen (Thermo Fisher Scientific, cat# R0551), 3 M sodium acetate (pH5.2, Quality Biological, cat# 351035721), ethyl alcohol (Thermo Scientific Richard-Allan Scientific, for in vitro diagnostic use), isopropyl alcohol (Thermo Scientific Richard-Allan Scientific, for in vitro diagnostic use), nuclease-free water (Cell Signaling Technology, cat# 12931S), mixture vortex (VWR, Analog Vortex Mixer), and centrifuge (Thermo Scientific, Legend Micro 21R). MagMAX Blood RNA Isolation Kit (ThermoFisher Scientific, Cat# AM1837), NucleoSpin for miRNA and RNA purification kit (Macherey-Nagel, Cat# 740971.50), and Plasma/Serum RNA Purification kit (Norgen Biotek Corp, Cat# 56100).

### Denaturing urea polyacrylamide gel and silver staining

Ten microliters of sample were mixed with an equal volume of gel loading buffer II (Thermo Fisher Scientific, cat# AM8546G), and the small RNA marker (Abnova, cat# R0007) was used. The mixtures were heated to 95 °C for 5 min to denature any secondary structure. Samples and markers were separated in 15% denaturing polyacrylamide gels (Invitrogen, cat# EC6885BOX) at 190 V for 50 min. Silver staining was performed using a SilverXpress Silver Staining Kit (Thermo Fisher Scientific, Cat# LC6100) according to the manufacturer’s instructions.

### Adaptor ligation, RT, and qPCR

The whole process of evaluating pfeRNA expression levels was similar to that we described before^4,7,17,25^. Specifically, the method includes adaptor ligation, RT, and QuantStudio PCR. An adaptor with both 5′ and 3′ modifications ligates only the 3′-end of sncRNA and enhances the ligation efficiency. For each ligation reaction, 5 µl total sncRNAs and 1 µl (2 µM) adaptor were ligated using single-strand truncated T4 RNA ligase 2 (New England Biolabs, cat# M0242 L) overnight at 16 °C, and the ligation reaction was terminated at 65 °C for 15 min. For RT, the SuperScript II First-Strand Synthesis System (Thermo Fisher Scientific, cat# 18064) and gene-specific reverse primers were used, and the total volume was 20 µl after RT. For QuantStudio PCR, a common reverse primer and primers specific for individual pfeRNAs were used. Each sample was tested in duplicate, and the total volume of each reaction was 20 µl. Amplification conditions were denaturation at 95 °C for 15 s (15 min for the first cycle), annealing at 60 °C for 20 s, extension at 72 °C for 20 s, and 40 cycles using a QuantStudio 3 machine (Applied Biosystem in Thermo Fisher Scientific). Each experiment was repeated three independent times. All primers and adaptors are listed in Supplementary Table S2.

## Supplementary Information

Supplementary Information.

## Data Availability

All data generated or analyzed during this study are included in this published article and its supplementary information files.
