# Supplementary material for: A TRIzol-based method for high recovery of plasma sncRNAs approximately 30 to 60 nucleotides
Source: Sci Rep. 2022 Apr 26;12:6778. doi: 10.1038/s41598-022-10800-0 (PMC9042852; doi:10.1038/s41598-022-10800-0)
Supplement: Supplementary file 1 — Supplementary Information. [file 41598_2022_10800_MOESM1_ESM.docx]

**Supplementary File**

This supplementary File includes Supplementary Figures 1 to 5, and Tables 1 and 2.


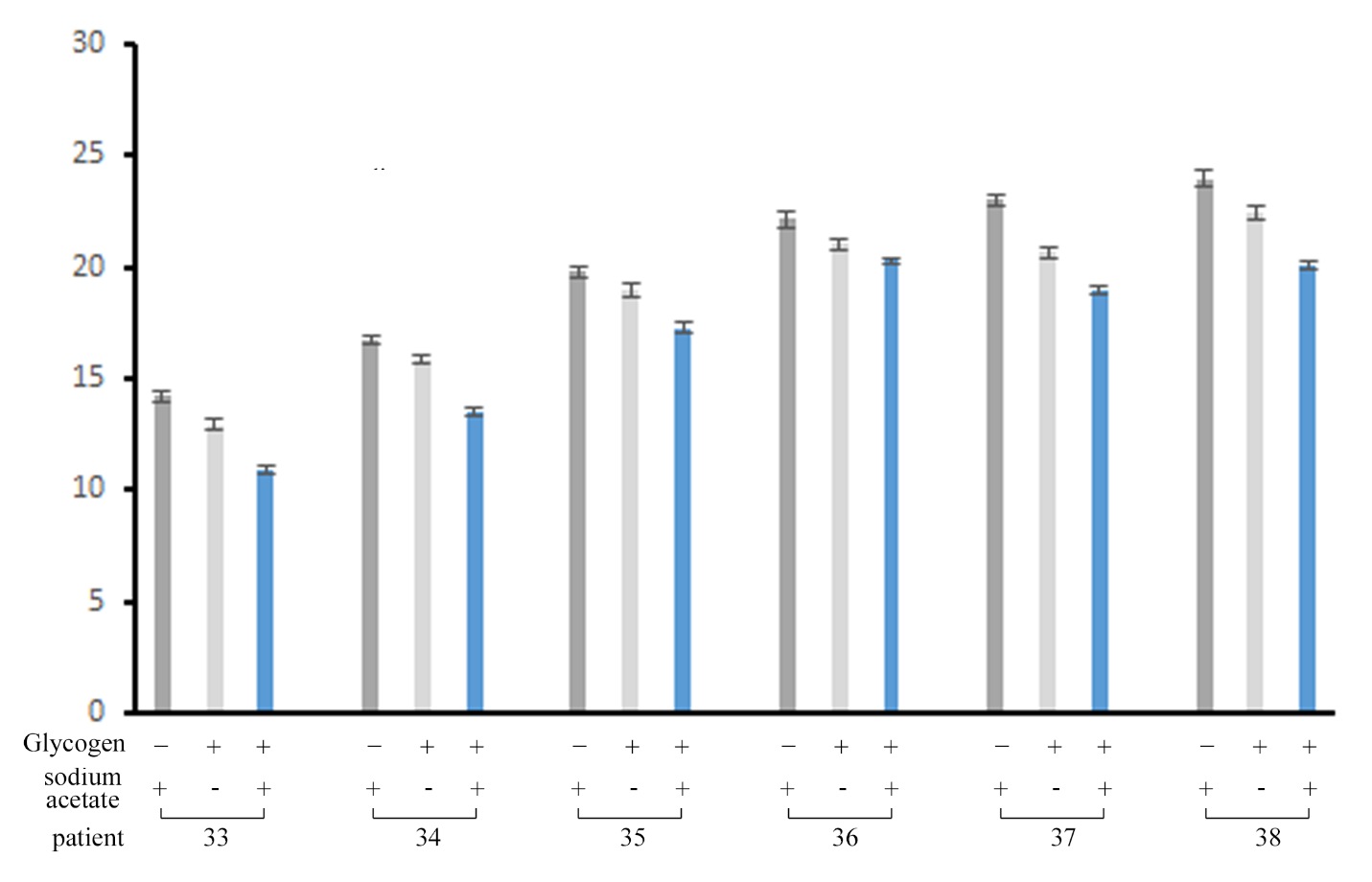


Supplementary Fig. 1. The specific CT values of pfeRNA from six clinical specimens by the TFP method with or without glycogen and sodium acetate, respectively.


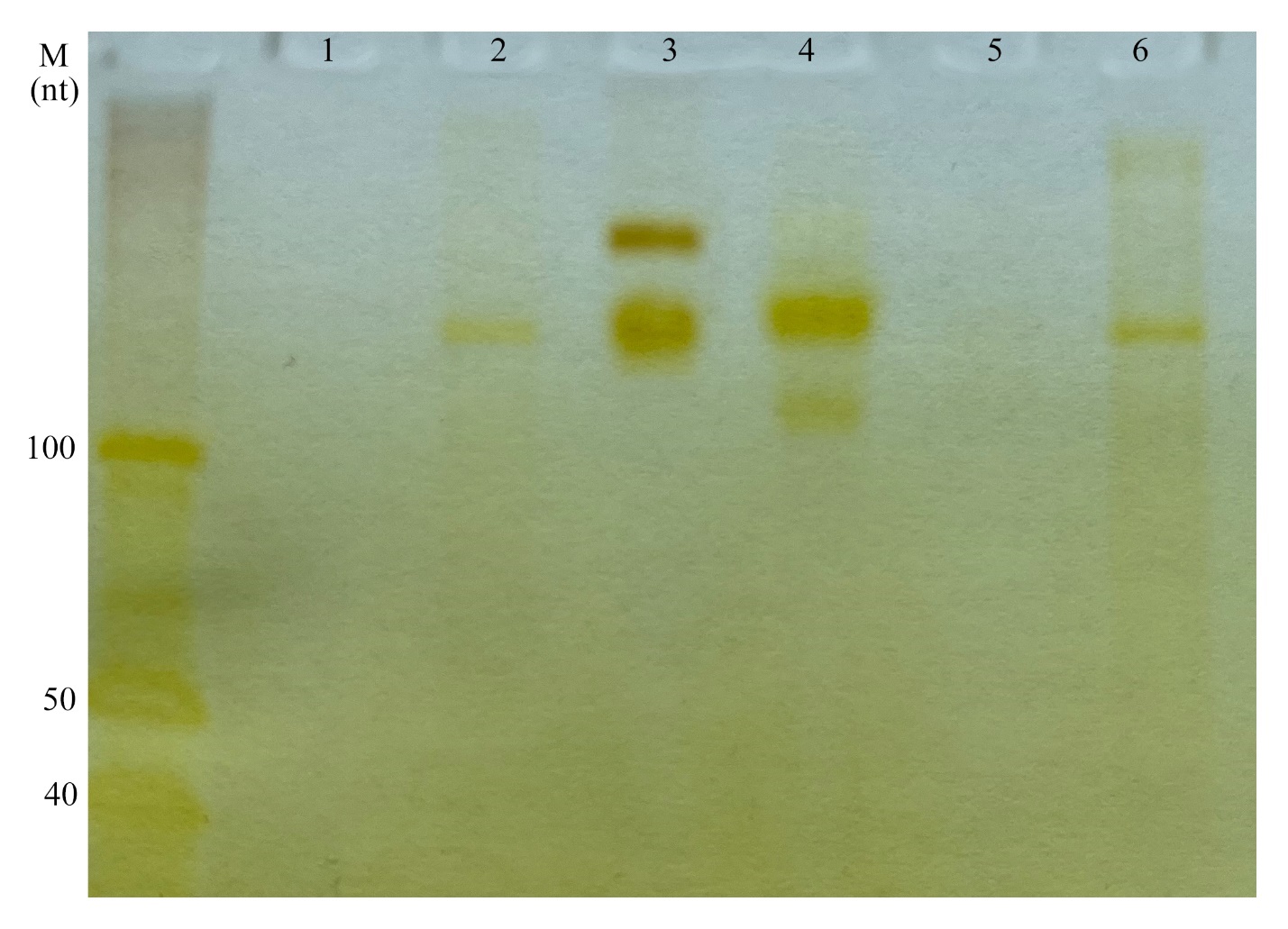


Supplementary Fig. 2. The extracted sncRNA distribution in the high-resolution gel was determined by silver staining. The plasma from the same patient was used for RNA extraction by different methods and kits, and the total recovered RNAs from 100µl plasma were used for gel resolution. Lane 1, 2, 3, 4, 5, and 6 showed sncRNA yields by TRIzol Reagent, TRIzol LS Reagent, MagMAX Blood RNA Isolation Kit from the ThermoFisher Scientific, TFP method, Plasma/Serum RNA Purification kit from Norgen Biotek Corp, and NucleoSpin for miRNA and RNA purification kit from Macherey-Nagel, respectively. This figure was cropped from Supplementary Fig. 7.


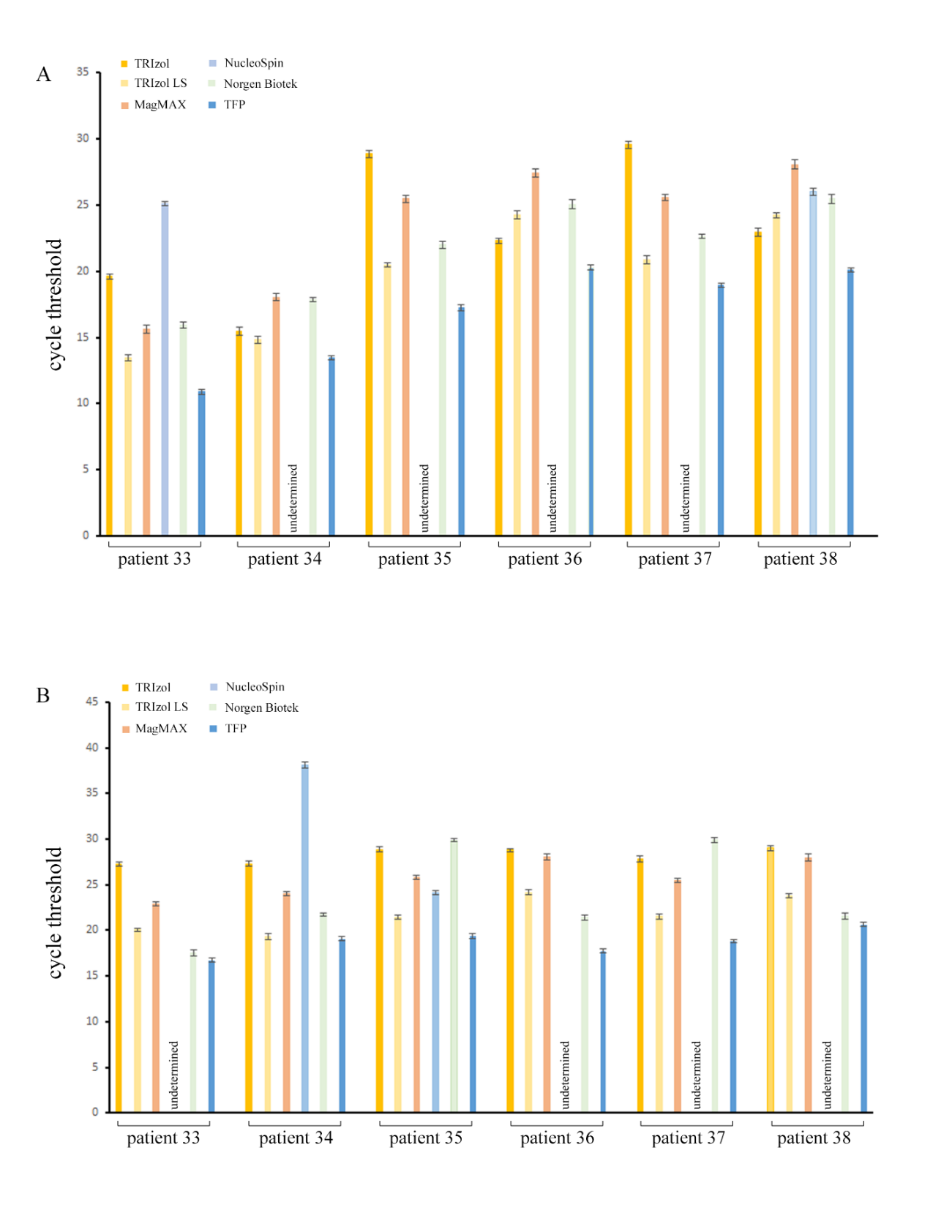


Supplementary Fig. 3. The specific CT values of pfeRNA of 34 nt (A) and 58 nt (B) from 6 clinical specimens by two methods based on organic extraction, three commercial kits, and the TFP method.


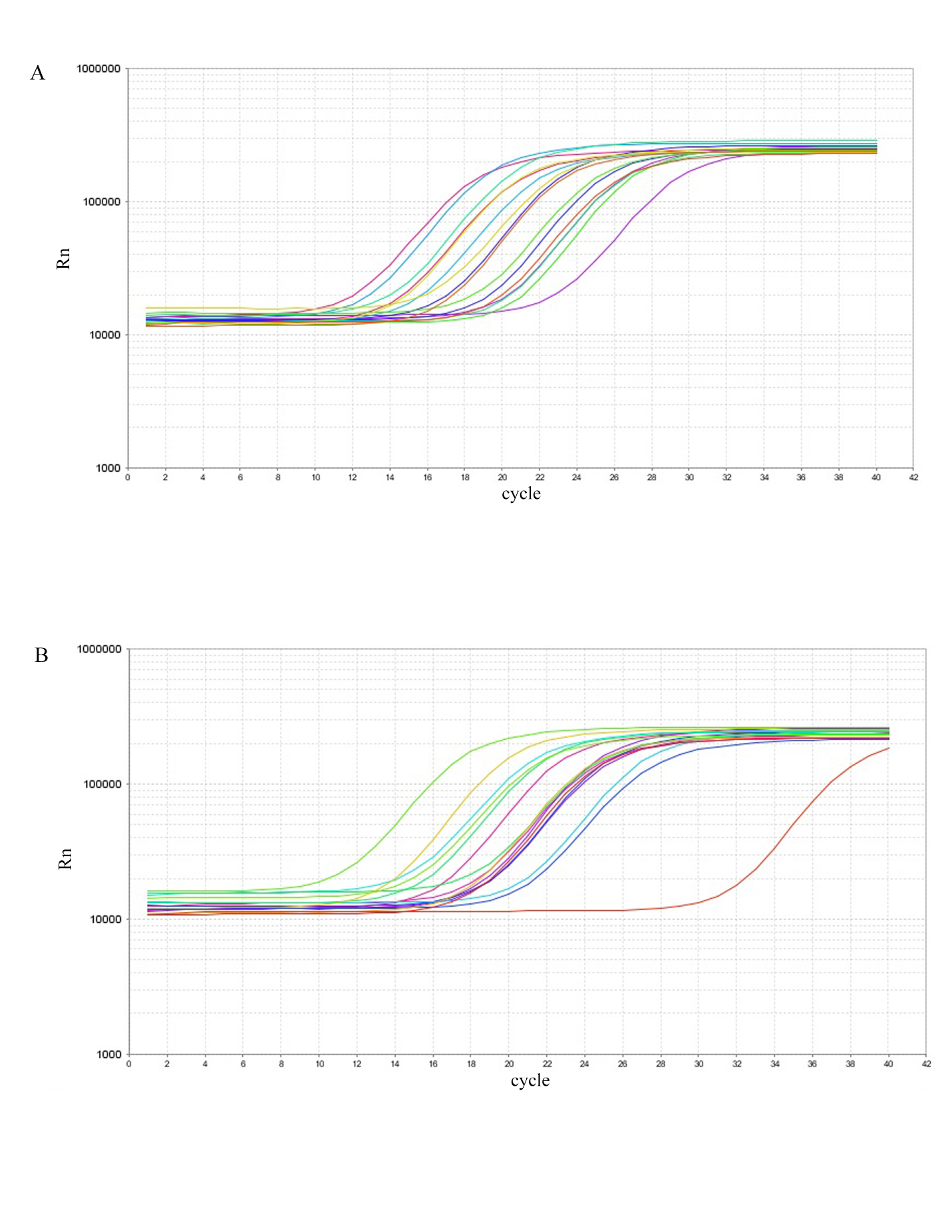
Supplementary Fig. 4. The amplification curves of targets recovered by the TFP method (A) and the commercial kit (B).


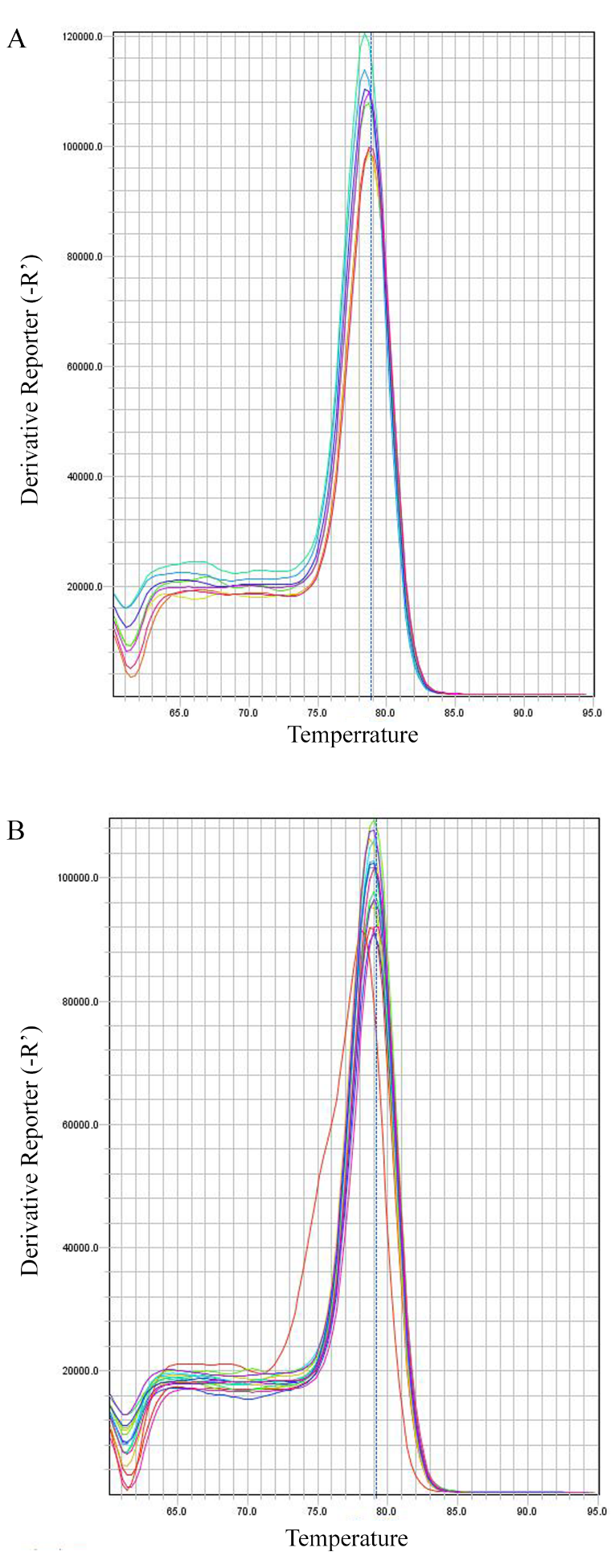
Supplementary Fig. 5. The melting curves of targets recovered by the TFP method (A) and the commercial kit (B).


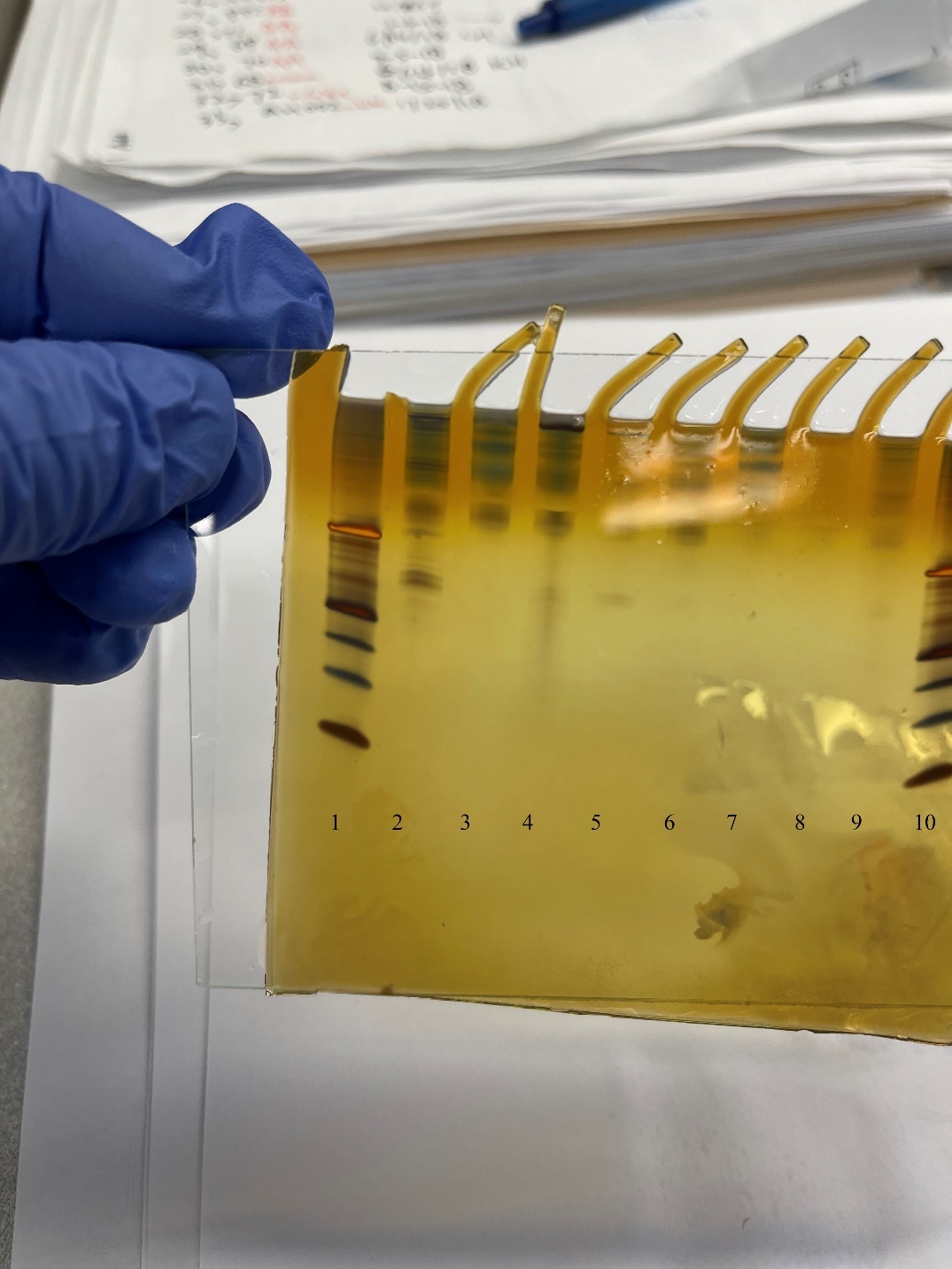


Supplementary Fig. 6. The original gel was used for Fig.2C. Lane 1 to Lane 3 were explained in the main text. Lanes 4, 5, and 9 showed sncRNA yields of three samples extracted by the TFP method, and Lanes 6, 7, and 8 showed sncRNA yields of three samples extracted by the commercial kit. The marker in Lane 10 is the same as that in Lane 1. No overexposure was used for the gel.


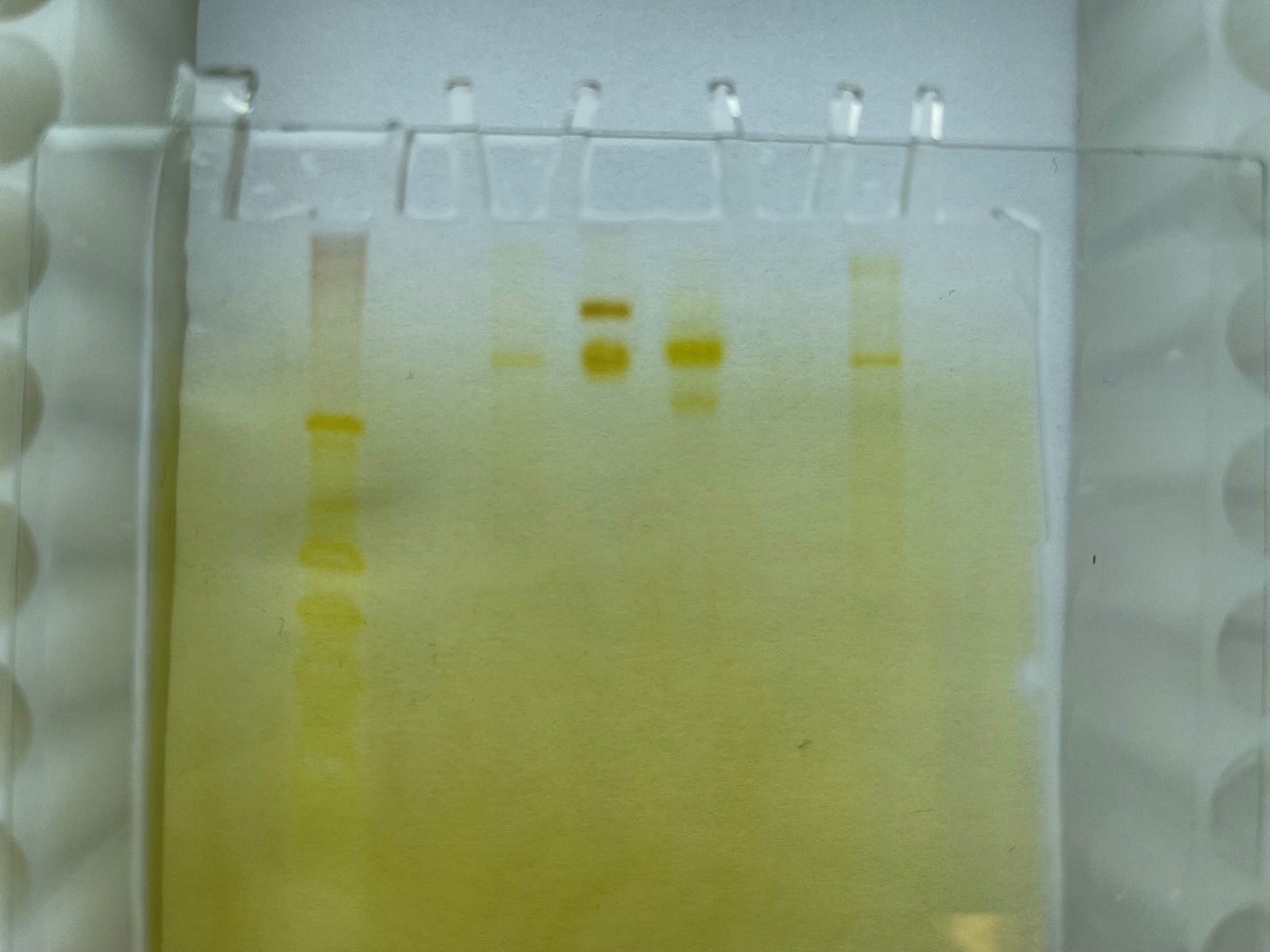


Supplementary Fig. 7. The original gel was used for Supplementary Fig. 2. All lanes were explained in the figure legend of Supplementary Fig. 2.

Supplementary Table S1. The clinical demographics of the patients.

|  |  |  |  |  |  |  |
| --- | --- | --- | --- | --- | --- | --- |
|  |  |  |  |  |  |  |
|  | Caucasian | Asian | African American | Latin | Mexican | Mix |
| Patient Numbers | 15 | 6 | 10 | 2 | 3 | 2 |
| Male | 9 | 3 | 7 | 2 | 2 | 2 |
| Female | 6 | 3 | 3 | 0 | 1 | 0 |
| Benign Nodules | 3 | 2 | 2 | 0 | 0 | 0 |
| NSCLC | 12 | 4 | 8 | 2 | 3 | 2 |
| smoker | 10 | 3 | 8 | 1 | 2 | 1 |
| non-smoker | 5 | 3 | 2 | 1 | 1 | 1 |

Supplementary Table S2. The specific sequences of primers and adaptor. RT (Reverse Transcription) and CRP (common reverse primer).

| name | sequences (5’ to 3’) | application |
| --- | --- | --- |
| 3' adaptor | rapp/5'-CTGTAGGCACCATCAAT-3'/3'ddc | ligation |
| gene-specific primer | CAAGCAGAAGACGGCATACGATTGATGGTGCCTACAG | RT |
| CRP | CAAGCAGAAGACGGCATACGA | qPCR |
| pfeRNA34nt F | TGGTTTTTCATATCATTGGTCGTGGT | qPCR |
| pfeRNA45nt F | CAACTTAACTTGACCGCTCTGACCA | qPCR |
| pfeRNA53nt F | AAGCACCCAACTTACACTTAGGAGA | qPCR |
| pfeRNA58nt F | ACTTACACTTAGGAGATTTCAACT | qPCR |
